# Individual- and Neighborhood-Level Predictors of Mortality in Florida Colorectal Cancer Patients

**DOI:** 10.1371/journal.pone.0106322

**Published:** 2014-08-29

**Authors:** Stacey L. Tannenbaum, Monique Hernandez, D. Dandan Zheng, Daniel A. Sussman, David J. Lee

**Affiliations:** 1 Sylvester Comprehensive Cancer Center, University of Miami Miller School of Medicine, Miami, Florida, United States of America; 2 Department of Public Health Sciences, University of Miami Miller School of Medicine, Miami, Florida, United States of America; 3 Department of Medicine, University of Miami Miller School of Medicine, Miami, Florida, United States of America; University General Hospital of Heraklion and Laboratory of Tumor Cell Biology, School of Medicine, University of Crete, Greece

## Abstract

**Purpose:**

We examined individual-level and neighborhood-level predictors of mortality in CRC patients diagnosed in Florida to identify high-risk groups for targeted interventions.

**Methods:**

Demographic and clinical data from the Florida Cancer Data System registry (2007–2011) were linked with Agency for Health Care Administration and US Census data (n = 47,872). Cox hazard regression models were fitted with candidate predictors of CRC survival and stratified by age group (18–49, 50–64, 65+).

**Results:**

Stratified by age group, higher mortality risk per comorbidity was found among youngest (21%), followed by middle (19%), and then oldest (14%) age groups. The two younger age groups had higher mortality risk with proximal compared to those with distal cancer. Compared with private insurance, those in the middle age group were at higher death risk if not insured (HR = 1.35), or received healthcare through Medicare (HR = 1.44), Medicaid (HR = 1.53), or the Veteran’s Administration (HR = 1.26). Only Medicaid in the youngest (52% higher risk) and those not insured in the oldest group (24% lower risk) were significantly different from their privately insured counterparts. Among 18–49 and 50–64 age groups there was a higher mortality risk among the lowest SES (1.17- and 1.23-fold higher in the middle age and 1.12- and 1.17-fold higher in the older age group, respectively) compared to highest SES. Married patients were significantly better off than divorced/separated (HR = 1.22), single (HR = 1.29), or widowed (HR = 1.19) patients.

**Conclusion:**

Factors associated with increased risk for mortality among individuals with CRC included being older, uninsured, unmarried, more comorbidities, living in lower SES neighborhoods, and diagnosed at later disease stage. Higher risk among younger patients was attributed to proximal cancer site, Medicaid, and distant disease; however, lower SES and being unmarried were not risk factors in this age group. Targeted interventions to improve survivorship and greater social support while considering age classification may assist these high-risk groups.

## Introduction

Colorectal cancer (CRC) is the second leading cause of cancer death for men and women combined in the U.S. with 142,820 estimated incident cases and 50,830 deaths in 2013 [1]. In the state of Florida in 2010, the age-adjusted incidence and mortality rates per 100,000 for CRC were 36.4 (95% Confidence Interval [CI] = 35.6–37.1) and 14.1 (95% CI = 13.7–14.6), respectively [2]. Adherence to screening guidelines leading to earlier detection has afforded patients long-term improvements in cancer-specific mortality risk [3], [4]. Moreover, there is a 90.1% five-year relative survival rate when CRC is detected at the localized stage of disease; however, these rates drop with invasive cancer diagnosed at regional and distant sites (69.2% and 11.7%, respectively) [5]. Healthy People 2020 objectives target CRC-specific mortality reduction from 17.0 per 100,000, the 2007 mortality rate, to 14.5 per 100,000 in 2020 [6]. Improved treatments, earlier detection and methods of prevention like removal of pre-cancerous polyps at time of screening procedures could effectively decrease the risk of death from CRC.

Survivorship of CRC may also depend on factors other than stage at diagnosis. Incidence of CRC [7], [8] and mortality [9] have been shown to differ by race and ethnicity. Moreover, improvements in CRC survival among Black CRC patients are attenuated when compared to that of White patients. Between 1992 and 2002 White patients had an annual decreased CRC mortality rate of 1.9% compared to a 0.8% decrease for Blacks over the same time period [9]. Survival may also be affected by other demographic characteristics such as age, gender, and socioeconomic status (SES), and by clinical characteristics, including treatments, cancer site within the colon, and comorbidities [10]–[12].

At the time of this publication, the authors were unable to identify any previous population-based study addressing all-cause survival after CRC diagnosis while simultaneously adjusting for the aforementioned particular demographic variables, clinical characteristics and comorbidities in both inpatients and outpatients 18 years of age and older with CRC. In contrast to previous studies, our research was novel in that we were able to incorporate CRC patients of a variety of age groups, stratifying by those with early-onset CRC while also including those of Medicare-beneficiary age, and those from statewide clinical practice locations [13], [14]. The data presented herein are also unique in that the state of Florida is inhabited by a multiracial, multiethnic, and economically diverse population of CRC survivors of all ages whose clinical information have been linked to high quality administrative and US Census sources. We therefore examined individual-level and neighborhood-level predictors of survival in CRC patients aged 18 and older who were diagnosed in Florida in order to identify high-risk groups for targeted clinical and social support interventions.

## Methods

Data were extracted from the Florida Cancer Data System (FCDS) for all cases of CRC incidence among Florida residents with age greater than or equal to 18 years, who were diagnosed between 2007 and 2011, and who had a valid 2010 census tract assignment based on geocoded addresses at the time of diagnosis (n = 47,872). In the state of Florida reside 19.6 million people; this is approximately 6% of the population of the US. The racial/ethnic breakdown of Florida is 57.0% White, 23.2% Hispanic, 16.6% Black, and 2.7% Asian [15]. The Hispanic population in South Florida primarily originates from the Caribbean, Central and South America, and Spain, potentially making the Florida population representative of the general Hispanic community [16]. The FCDS is a statewide, population-based cancer incidence registry created by the State of Florida Department of Health in 1978, and operated by the Sylvester Comprehensive Cancer Center at the University of Miami Leonard M. Miller School of Medicine (Miami, FL) with support from the Florida Department of Health and from the Centers for Disease Control and Prevention and National Program for Cancer Registries. FCDS was granted a gold standard rating as it collects 98% of all incident cancers in Florida.

### Outcome Variable

Mortality was our outcome variable and was defined as time from the index date of diagnosis to the date of death or the date of last patient follow-up. The FCDS performs passive follow-up of patient status through a series of linkages with the Florida Office of Vital Statistics as well as the National Death Index. The most recent linkage with the Florida Office of Vital Statistics included deaths through the year 2011. Therefore, the last date of passive follow-up was set as December 31^st^, 2011. Follow-up interval was the time between the index date (date of cancer diagnosis) to the date of death; or from index date until December 31, 2011 for those who survived. If there was no healthcare encounter during this time period it was assumed that the patient was still alive by the end date of this study. FCDS abstracters provide a minimum of annual updates to patient treatment and vital status obtained by review of the Social Security Death Index, obituaries and inquiry to patients, family members, and/or outpatient clinics.

### Demographic Characteristics

Demographic, tumor, and treatment variables were grouped into larger categories. Race and ethnicity variables were combined to tabulate mutually exclusive groups by non-Hispanic and Hispanic classifications, which included non-Hispanic White, non-Hispanic Black, Hispanic, non-Hispanic other, and unknown if Hispanic. Age was included as a continuous variable in the overall sample and within the three age groups of 18–49, 50–64, and 65+ years. Sex was grouped by male or female. Primary payer at diagnosis was grouped into private insurance, Medicaid, Medicare, Military/Veterans Administration (VA), not insured, insurance not otherwise specified (NOS), or unknown. The FCDS does not derive data from the VA Medical Center; data for those veterans included in the present study relate to those members who obtained health care services outside of the VA system. Marital status was broken down by married, single, divorced/separated, widowed, or unknown.

### Socioeconomic Status (SES) Measures

SES measures were obtained from the U.S. Census Bureau data files at the census tract level using the pooled 2006–2010 American Community Survey data and were linked with cancer records using geocoded residential locations at the time of patient diagnosis. Included in the census linkage was the percent of the population whose income in the 12 months preceding diagnosis was below the poverty level. The neighborhood poverty level (SES) was categorized as follows: lowest (≥20%), middle low (≥10 and <20%), middle high (≥5 and<10%), and highest (<5%) SES based on the state quartile distributions for percentage of the neighborhood living in poverty.

### Comorbidity

For the corresponding diagnosis years, the cancer data were linked with Florida hospital and outpatient discharge data from the Agency for Health Care Administration using patient social security number and date of birth in a deterministic matching process. Data on comorbid status were retained using any reported secondary diagnosis International Classification of Diseases (ICD)-9-CM value for inpatients and outpatients, which were then summarized by comorbid category. Additional comorbidities were also derived from diagnosis-related group (DRG) codes for inpatient hospital stays. Comorbidity Software, Version 3.7 from the Health Cost and Utilization Project (an endeavor sponsored by the Agency for Healthcare Research and Quality) was utilized to determine Elixhauser comorbidity groups [17]. A binary indicator was created for each Elixhauser comorbidity category, with “one” indicating patient had in-patient or out-patient visit(s) related to this comorbidity category. Multiple visits related to the same category were only counted once. A comorbidity index was then calculated for each patient by summarizing the Elixhauser comorbidity category indicators. Comorbidity categories related to cancer or tumors were excluded from the summary count.

### Tumor Characteristics

Primary cancer site data were coded according to the International Classification of Diseases for Oncology in use at the time of diagnosis, converted to the third edition [18]. Colorectal classification included all sites coded C18.0 through C20.9 with the exception of the appendix (C18.1) and Large Intestine NOS (C18.8–C18.9). Colorectal subsite locations were categorized into proximal (cecum, ascending colon, hepatic flexure, and transverse colon), distal (splenic flexure, descending colon, and sigmoid colon), and rectum (rectosigmoid junction, rectum). Staging was derived from the 2000 Surveillance Epidemiology and End Results coding systems [19], of which our analysis included the following staging categories: *in situ*, localized, regional and distant.

### Treatment Factors

All surgical, radiation, and chemotherapy treatment codes were collapsed into three categories; treatment received, treatment not received, and unknown if treatment was received.

### Statistical Analysis

Patient records were de-identified prior to analysis. Descriptive statistics of above mentioned demographic and clinical factors were analyzed for the CRC cases in Florida. Cox proportional hazard regressions were performed first for each demographic and clinical factor individually in univariate models and then with all demographic and clinical factors in one model to determine the association with all-cause mortality in CRC patients while controlling for all covariates; only multivariable models are presented in the table. Multivariable models were also performed with stratification by age groups (18–49, 50–64, and 65+). No interactions were found among main predictor variables. All statistical analyses were performed using SAS 9.3 (SAS Institute, Cary, NC) software.

## Results

### Demographic Characteristics

The study sample consists of 47,872 CRC patients who were diagnosed between 2007 and 2011 in Florida of whom 52.1% were male, 65.3% were 65 years and older, 72.9% were non-Hispanic White, and 53.4% were married (Table 1). The mean follow-up time for all subjects was 2.0 person-years (731 days). The majority of patients underwent surgery (84.7%), 28.5% received chemotherapy and 10.3% had radiation therapy. Thirty-one percent of patients were from the highest quartile SES neighborhood, 28.5% were from middle-high, and 15.0% were from the lowest quartile. In terms of the Elixhauser comorbidity index, 28.5% had no comorbidities, 15.6% had one, 16.3% had two, and 39.5% had three or more, with the maximum number of comorbidities of 14 in any single individual.

**Table 1:** Descriptive characteristics of the sample from the Florida Cancer Data System and the Agency for Health Care Administration datasets (2007–2011).

Characteristic		N	%
Sex			
	Male	24,921	52.06
	Female	22,951	47.94
Mean age in years (SD)	69.1	(13.5)	Range 18–104
Race/Ethnicity			
	NH White	34,896	72.89
	NH Black	5,061	10.57
	Cuban	1,386	2.90
	Puerto Rican	231	0.48
	Mexican	96	0.20
	Other Hispanics	5,049	10.55
	NH Other	842	1.76
	Unknown Ethnicity	311	0.65
Stage			
	In Situ	2,499	5.22
	Localized	17,728	37.03
	Regional	16,534	34.54
	Distant	8,097	16.91
	Unknown	3,014	6.30
Insurance Status			
	Uninsured	1,935	4.04
	Private	12,049	25.17
	Medicare	27,504	57.45
	Medicaid	2,547	5.32
	Military/Veteran	739	1.54
	Insurance NOS	2,095	4.38
	Unknown	1,003	2.10
Marital Status			
	Single	7,188	15.02
	Married	25,546	53.36
	Divorced/Separated	4,812	10.05
	Widowed	8,967	18.73
	Unknown	1,359	2.84
Tumor Site			
	Distal Colon	12,839	26.82
	Proximal Colon	21,581	45.08
	Rectum	13,452	28.10
Surgery			
	Yes	40,537	84.68
	No	6,912	14.44
	Unknown	423	0.88
Chemotherapy			
	Yes	13,633	28.48
	No	33,492	69.96
	Unknown	747	1.56
Radiation			
	Yes	4,934	10.31
	No	42,622	89.03
	Unknown	316	0.66
SES*			
	Highest	14,911	31.15
	Middle high	13,639	28.49
	Middle low	12,093	25.26
	Lowest	7,229	15.10
Number of Major Comorbidities			
	0	13,665	28.54
	1	7,484	15.63
	2	7,809	16.31
	3	6,749	14.10
	4	4,896	10.23
	5	3,125	6.53
	6	1,847	3.86
	7	1,145	2.39
	8	618	1.29
	9	303	0.63
	10	118	0.25
	11	68	0.14
	12	33	0.07
	13	8	0.02
	14	4	0.01

*SES = socioeconomic status defined by 4 categories of lowest (≥20%), middle-low (≥10 and <20%), middle high (≥5 and<10%), and highest (<5%) SES based on percentage of the neighborhood living in poverty; NH = non-Hispanic; NOS = not otherwise specified.

### Demographic Predictors

In the multivariable survival analysis model for all-cause mortality, females had a Hazard Ratio (HR) of 0.85 compared to males (Table 2). This sex disparity remained when we stratified by age group. Hispanics compared to non-Hispanic Whites had a survival benefit with a HR of 0.85 but non-Hispanic Blacks were not significantly different from non-Hispanic Whites (p = 0.81). Stratifying by age group, none of the race/ethnicities in the youngest age group were significantly different compared to Whites, however, Hispanics in the two older groups were at lower mortality risk (HR = 0.75 in 50–64 and 0.89 in 65+) while only non-Hispanic Blacks in the 50–64 age group were at lower mortality risk (HR = 0.89).

**Table 2:** Multivariable analysis to predict mortality from CRC stratified by age group from the Florida Cancer Data System and the Agency for Health Care Administration datasets (2007–2011).

		Age Groups		
	Overall	18–49 years	50–64 years	65+ years
		N = 4,077	N = 12,560	N = 31,235
**Prognostic factors**	HR (95% CI)	HR (95% CI)	HR (95% CI)	HR (95% CI)
**Sex**				
Male	1.00	1.00	1.00	1.00
Female	0.85 (0.82–0.88)^***^	0.81 (0.70–0.93)^**^	0.87 (0.80–0.94)^**^	0.83 (0.80–0.86)^***^
**Age at diagnosis**	1.03 (1.04–1.04)^***^	1.01 (0.99–1.02)	1.01 (1.00–1.02)*	1.05 (1.05–1.05)^***^
**Race/ethnicity**				
NH White	1.00			
Hispanic	0.85 (0.81–0.89)^***^	1.05 (0.86–1.28)	0.75 (0.66–0.85)^***^	0.89 (0.83–0.94)^***^
NH Black	0.99 (0.94–1.05)	1.17 (0.98–1.40)	0.89 (0.80–0.99)*	1.02 (0.94–1.09)
NH Other	0.73 (0.63–0.84)^***^	0.52 (0.27–1.00)	0.86 (0.65–1.13)	0.73 (0.60–0.88)^**^
Unknown	0.60 (0.45–0.81)^**^	0.32 (0.08–1.29)	0.61 (0.30–1.23)	0.63 (0.46–0.88)^**^
**Marital Status**				
Married	1.00	1.00	1.00	1.00
Divorced/Separated	1.22 (1.15–1.30)^***^	1.11 (0.88–1.40)	1.18 (1.06–1.32)^**^	1.28 (1.19–1.38)^***^
Single	1.29 (1.22–1.35)^***^	1.11 (0.94–1.31)	1.23 (1.11–1.35)^***^	1.26 (1.18–1.34)^***^
Widowed	1.19 (1.14–1.25)^***^	0.91 (0.94–1.31)	1.14 (0.94–1.37)	1.13 (1.08–1.19)^***^
Unknown	1.00 (0.90–1.11)	0.91 (0.61–1.36)	1.22 (0.97–1.54)	0.93 (0.83–1.06)
**Cancer Site**				
Distal Colon	1.00			
Proximal Colon	1.03 (0.99–1.07)	1.23 (1.03–1.47)*	1.28 (1.16–1.40)^***^	0.96 (0.92–1.01)
Rectum	0.98 (0.93–1.03)	0.89 (0.73–1.08)	0.99 (0.89–1.10)	0.99 (0.94–1.05)
**Stage**				
Localized	1.00	1.00	1.00	1.00
In Situ	1.09 (0.98–1.21)	0.78 (0.36–1.71)	0.82 (0.59–1.13)	1.19 (1.06–1.32)
Regional	1.83 (1.75–1.92)^***^	2.72 (2.02–3.67)^***^	2.61 (2.27–3.00)^***^	1.72 (1.63–1.81)^***^
Distant	6.07 (5.77–6.39)^***^	11.94 (8.92–15.98)^***^	10.86 (9.47–12.46)^***^	5.84 (4.85–5.45)^***^
Unknown	1.82 (1.69–1.96)^***^	4.07 (2.79–5.94)^***^	2.76 (2.28–3.35)^***^	1.60 (1.47–1.74)^***^
**Insurance**				
Private	1.00	1.00	1.00	1.00
Not Insured	1.22 (1.11–1.35)^***^	1.17 (0.93–1.48)	1.35 (1.20–1.53)^***^	0.76 (0.60–0.97)*
Medicare	1.03 (0.98–1.08)	1.30 (0.95–1.78)	1.44 (1.28–1.63)^***^	1.00 (0.93–1.07)
Medicaid	1.42 (1.30–1.54)^***^	1.52 (1.25–1.85)^***^	1.53 (1.36–1.72)^***^	1.11 (0.95–1.29)
Veterans	1.19 (1.03–1.39)^***^	1.53 (0.92–2.53)	1.26 (1.01–1.57)*	1.10 (0.88–1.38)
Insurance NOS	1.03 (0.94–1.14)	1.29 (1.00–1.66)*	1.00 (0.86–1.17)	1.00 (0.86–1.16)
Unknown	1.36 (1.22–1.52)^***^	1.11 (0.73–1.70)	1.24 (0.99–1.56)	1.41 (1.22–1.62)^***^
**Surgery**				
No	1.00	1.00	1.00	1.00
Yes	0.42 (0.40–0.44)^***^	0.39 (0.33–0.47)^***^	0.42 (0.38–0.46)^***^	0.43 (0.41–0.45)^***^
Unknown	0.81 (0.68–0.96)*	1.77 (0.85–3.69)	0.89 (0.62–1.30)	0.80(0.65–0.99)^***^
**Chemotherapy**				
No	1.00	1.00	1.00	1.00
Yes	0.66 (0.63–0.69)^***^	0.72 (0.61–0.85)^**^	0.66 (0.60–0.72)^***^	0.65 (0.61–0.69)^***^
Unknown	0.93 (0.80–1.07)	0.49 (0.21–1.15)	1.04 (0.73–1.42)	0.94 (0.80–1.10)
**Radiation**	1.00			
No	1.00	1.00	1.00	1.00
Yes	0.93 (0.86–0.99)*	1.10 (0.87–1.38)	1.02 (0.89–1.17)	0.89 (0.81–0.97)*
Unknown	1.68 (1.34–2.10)^***^	1.58 (0.60–4.21)	1.04 (0.73–1.47)	1.56 (1.20–2.02)^**^
**SES^¥^**				
Highest	1.00	1.00	1.00	1.00
Middle-high	1.04 (1.00–1.09)	1.03 (0.84–1.27)	1.05 (0.94–1.18)	1.04 (0.99–1.09)
Middle-low	1.13 (1.08–1.19)^***^	1.08 (0.88–1.32)	1.17 (1.05–1.30)^**^	1.12 (1.06–1.18)^***^
Lowest	1.19 (1.13–1.26)^***^	1.11 (0.89–1.39)	1.23 (1.09–1.39)^**^	1.17 (1.10–1.25)^***^
**Comorbidities (count)**	1.15 (1.15–1.16)^***^	1.21 (1.17–1.24)^***^	1.19 (1.17–1.21)^***^	1.14 (1.13–1.15)^***^

*P<0.05; **P<0.01; ***P<0.0001; ^¥^SES = socioeconomic status defined by 4 categories of lowest (≥20%), middle-low (≥10 and <20%), middle high (≥5 and<10%), and highest (<5%) SES based on percentage of the neighborhood living in poverty; NH = non-Hispanic; NOS = not otherwise specified.

Age was a statistically significant predictor of mortality risk; there was a 3% higher mortality risk with annual increase in age. When we stratified by age group, the patients in the youngest group had no increase in mortality risk with age. However, in the middle and oldest age groups there was an increase in mortality risk with each increase in age (1% and 5%, respectively).

Married patients were significantly better off than divorced/separated (HR = 1.22), single (HR = 1.29), or widowed (HR = 1.19) patients. Age stratified models revealed that compared to married patients, divorced/separated and single patients in the two older groups and widowed patients 65+ had a higher risk of death. This phenomenon was not true for those in the youngest group.

Compared to those with primary payer at diagnosis of private insurance, those uninsured had 1.22 fold increased risk of mortality, those with Medicaid had a1.42 fold higher mortality risk, and Military/Veterans had a 1.19 fold higher mortality risk; but those with Medicare (P = 0.29) were no longer worse off than privately insured. In age-stratified models and compared with private insurance, those in the middle age group were at higher risk of death if not insured (HR = 1.35), had Medicare (HR = 1.44), had Medicaid (HR = 1.53), or had VA (HR = 1.26). Only Medicaid in the youngest age group (52% higher mortality risk) and those not insured in the oldest age group (24% lower mortality risk) were significantly different from their private insurance counterparts.

### Socioeconomic Status

Relative to those living in high SES neighborhoods at the time of diagnosis, those living in the two lowest SES neighborhoods had a survival disadvantage (HR = 1.13) and (HR = 1.19), respectively. However, middle high SES was not different from highest SES (P = 0.06). In the age stratified model only those in the youngest age group showed no difference in mortality risk regardless of SES group membership. For the two older age groups there was a higher mortality risk among the two lowest SES groups (1.17- and 1.23-fold higher in the middle age group and 1.12- and 1.17-fold higher in the older age group) compared to the highest SES.

### Comorbidity

There was a 15% higher risk of death per additional Elixhauser comorbidity group suffered by patients in the multivariable analysis. When stratified by age group, a higher mortality risk per comorbidity was 21% in the youngest group, 19% in the middle group, and 14% in the oldest age group.

### Tumor Characteristics

In the multivariable model, cancer site of proximal colon was no different than the referent distal colon with respect to survival (P = 0.19). In age stratified models, the two younger age groups had a higher mortality risk with proximal cancer compared to those with distal cancer (1.23-fold higher in the youngest and 1.28-fold higher in the middle age group).

As stage of disease became more advanced, survival time shortened in regional and distant. The age stratified analyses revealed that compared to those in the middle and oldest age groups, those in the youngest age group had the highest mortality risk from regional (2.72 times greater risk) and distant disease (11.94 times greater risk) when comparing to localized stage. In the oldest age group, those with regional or distant disease had 1.72-fold and 5.84-fold higher risk, respectively, than those with localized stage.

### Treatment Factors

In the multivariable model, having treatments imparted a beneficial outcome compared to not being treated. This remained true in the age stratified analysis for those treated with surgery and chemotherapy. However, only the oldest group had a survival advantage among those receiving radiation treatments while no difference in mortality was found in the two younger groups.

## Discussion

In this population-based study we looked at individual- and neighborhood-level predictors of CRC mortality in the demographically diverse state of Florida. We found that significant predictors of CRC mortality included marital class, primary payer at diagnosis, neighborhood socioeconomic category, comorbidity count, and tumor characteristics. Age stratifications confirmed the value of surgical and chemotherapeutic regimens for all age groups, with the advantage conferred by private insurance disappearing in the 65 and older age group. The disadvantage presented by lower SES persisted into the advanced age category. Though survival was worse in all ages with comorbidity, this effect was greatest in those less than 50 years of age.

### Demographic Characteristics

Marital status was a predictor of mortality in our study in that those who were not married were at greater mortality risk compared with those who were married. This may be true because married couples are more likely to undergo screening with their primary care physician or at clinics compared with those who are not married [20], and are more likely to be compliant with treatment recommendations [21]. Those patients who live alone have worse survival, likely related to the fewer treatments that these colon cancer patients undergo [22]. This survival benefit for married patients has been seen not only in CRC, but also with other cancers [23]. Although other investigators found similar results as we did for CRC, they only compared the broader category of “married” to “not married” without further delineation of participants’ unmarried status [24]. Our study was unique in that we differentiated “not married” as single, divorced/separated, and widowed as these subgroups are very different from one another. We found that when adjusted for all other factors and compared with married, the worst survival was observed for single individuals, divorced/separated and widowed patients in the oldest age group and single or divorced/separated in the middle age group. In accordance with our findings, married and divorced/separated CRC patients have greater survival benefit than single patients [25]. However, this was not true for the younger age group which suggests that marital class as a risk factor operates differently for older and younger individuals. Our findings also suggest that clinicians should encourage unmarried older patients to seek social support services following a diagnosis with CRC.

We found that primary payer at diagnosis is associated with survival of CRC patients. Compared to those with private insurance, those with Medicaid showed a 42% increase in mortality in the fully adjusted model. Other studies have found similar results for patients with Medicaid [24], [26]. The worse mortality may be explained by diminished access to care in those receiving Medicaid; Medicaid may also be a surrogate marker for chronic poverty which is its own barrier to treatment compliance [27]. Another consideration, however, is that some of our Medicaid observations may be due to confounding with uninsured individuals who acquire emergency Medicaid upon diagnosis of late stage CRC due to lack of access to care and then they subsequently have poorer outcomes.

This survival detriment in comparison to private patients disappeared in the 65 years and older age category, where eligible patients also receive Medicare. However, patients in the 50–64 year age group, i.e., before being age-eligible for Medicare, had a higher mortality risk than those 65+ when comparing primary payers at diagnosis with private insurance. Patients receiving Medicare below the age of 65 are likely a sicker population group. These findings are unique in that we were able to determine differences by primary payer in those less than Medicare-eligible ages, a limitation with SEER-based Medicare analyses.

Patients who were uninsured or receiving VA care in our series also experienced worse survival than those with private insurance. This is in contrast to the findings of other investigators showing that cancer survival in VA patients cared for within the VA system was better than in non-VA patients [28]. As FCDS does not collect information for patients using only the VA system, our findings must be interpreted with caution. Any CRC patient in our series claiming Military/Veterans as a primary payer must have been seeking care outside of the VA system. It has been shown that patients enrolled in dual systems of care, that is both VA and Medicare, may be worse off than those enrolled in either the VA or Medicare alone [29]. The rationale underlying this finding is unclear, but patients seeking care from multiple locations could potentially be sicker or have disease considered untreatable according to guidelines. Unexpectedly, we found a survival advantage in uninsured older individuals compared to their older counterparts with private insurance; the reason behind this finding is counterintuitive and may need further investigation.

Relative to non-Hispanic Whites in our study, a protective effect was seen in Hispanics and this effect remained for the two older age groups, but not among the youngest group. Other studies have found conflicting results [30], [31]. The better survival observed in our series may be related to the so-called Hispanic Paradox, and much work is needed to elucidate the roles of migration, acculturation, and social support structures in potentially explaining these survival differences.

### Socioeconomic Status

Our findings indicate that there is an effect of neighborhood poverty on survival from CRC; this was true for the two older age groups among those living in the lowest SES neighborhoods. Compared to the highest SES, those living in neighborhoods with lower SES had incrementally worse survival adjusting for disease stage and primary payer at diagnosis. These findings corroborate population-based Connecticut cancer registry results in that those living in the poorest census tracts had a higher mortality risk compared with their higher income counterparts [12]. Similar results have been well-documented by other state registries [10], [32]. SEER data demonstrate that lower SES and lack of treatments for colon cancer decrease survival, particularly among Black patients [33]. However, our data are unique in that we capture younger age groups than the SEER-Medicare data. These findings suggest that social class membership matters in terms of survival for those 50 years and older even when accounting for race/ethnicity, comorbidities, and stage at diagnosis. Moreover, approximately 40% of our sample lived in the most impoverished neighborhoods (lowest or middle low SES). This supports the need for additional targeted efforts of outreach to these communities in need, such as community-trained patient navigators, who have previously been successful in improving colon cancer screening adherence rates among low SES groups as well as treatment satisfaction for those uninsured who were diagnosed with CRC [34], [35]. Community health workers have also been valuable in providing outreach and education regarding CRC among the underserved [36].

### Comorbidity

The number of categories of comorbidity in patients with CRC had a profound effect on survival in our study. The risk of death increased 15% for each additional Elixhauser comorbidity, with the youngest patients having the highest risk (21%) and the oldest patients having the smallest risk (14%) per comorbidity. We included comorbidities in a dose-response fashion in our study because comorbidity is a known independent risk factor for CRC mortality [37]. Our findings are in line with other studies that show the effect of higher comorbidity and worsening of survival [38]. Robbins et al. found an association between comorbidity and insurance; uninsured, Medicaid, and Medicare insured had higher comorbidity levels [14]. An increase in comorbidity decreases survival, predominantly in patients with early stage CRC [39]. This finding is consistent with previous reports that the presence of comorbidity differentially affects the prognosis of those groups with longer survival (that is, earlier stage cancers) [40]. The topic of comorbidity in cancer raises many interesting questions [41]. In particular, the influence of comorbidities on cancer-specific or overall mortality is important; our series measured overall mortality with comorbidity being a strong predictor. Also unclear are the roles comorbid conditions play in an individual’s ability to complete prescribed anti-neoplastic treatments due to toxicity or intolerability [42]–[44]. And can these comorbidities identified in retrospective series serve as surrogate markers for other factors, like lifestyle choices or tumor biology? [41]. With these findings in mind, special attention should be given to CRC patients with numerous comorbidities as they are at higher risk for death regardless of age, but most pronounced in the youngest.

### Tumor Characteristics

Although we did not find cancer site in the whole sample to be a significant predictor of mortality risk, our novel finding was that proximal cancers were associated with greater mortality risk among those 18–64 years of age. This is an important finding in light of the fact that colonoscopy has diminished impact in attenuating mortality for proximal colon cancer when compared to distal cancers [45], and because guidelines for screening do not include those below the age of 50 [46]. SEER-Medicare data are, by design, limited to those 65 years and older. To our knowledge this is the first study to look at cancer site in younger age groups while controlling for demographic, other tumor characteristics, treatments, and comorbidities.

Our findings confirm that when potential confounding predictors are accounted for, late stage diagnosis of CRC is alarmingly deadly; patients were at six times the risk of death if diagnosed with distant disease. Le et al. found 34.5 times the risk of death at the latest stage but unlike our study, these investigators did not control for insurance, comorbidities, and marital status [10]. Robbins et al. found 21.6 times the risk of death but treatment, race/ethnicity, marital status, cancer site, and gender were not controlled for in their model [14]. Our study was also unique in that by stratifying by age group, we discovered that stage was a greater predictor in the younger age group. This observation highlights the importance of identifying and intervening upon modifiable risk factors for CRC in this younger population who are not captured in the current guidelines of screening as prevention for average risk individuals. The rising national incidence of CRC in Americans under age 50 makes this finding immediately relevant to clinical care [47].

Besides being diagnosed at later stage, our findings suggest that individuals requiring more medical attention are older individuals with more comorbidities, who are unmarried, uninsured, and living in lower SES neighborhoods; for younger individuals, increased attention may be warranted for those with proximal colon cancer, receiving Medicaid, and with more comorbidities. Moreover, it appears that increased quality of care and access to care are needed for patients with Medicaid, uninsured, or VA patients who seek care outside the VA system. The findings reinforce the supreme importance of increasing screening to prevent cancer or identify cancers at early stages, particularly for those groups at higher risk.

These observations also underline the importance of tools to identify patients with increased mortality in order to improve survivorship through intervention. A recent Institute of Medicine report supported the value of collecting cancer comorbidity data while recording patient-reported outcomes and health behaviors in order to create and track metrics for patient-centered, high quality cancer care [48]. Preliminary success with tools for colon cancer survivorship has been demonstrated through dissemination of National Comprehensive Cancer Network (NCCN) guidelines [49]. These tools promote the messages of oncologic societies like American Society of Clinical Oncology and NCCN for CRC treatment; adherence to these treatment guidelines is associated with improved survival in CRC patients and this should be encouraged among clinicians [50], [51]. Some evidence exists to improve survivorship through the creation of post-cancer care plans [52]; however, relatively few National Cancer Institute-designated cancer centers provide survivorship care plans to their CRC patients [53]. Surveillance colonoscopy is also underutilized in this high-risk group in danger of metachronous colonic dysplasia [54]. Some have recommended the creation of multi-disciplinary teams to improve uptake of treatments [55]. Finally, unknown variables like biology or cultural acceptance of treatment paradigms may affect cancer survivorship, as survival gaps for Blacks are not closing, despite controlling for stage and treatment variables [56]. There are clearly many understudied and unexploited opportunities for intervention to improve cancer survivorship.

There were several limitations to our study. We did not have information on CRC screening histories. The FCDS and AHCA databases do not have individual-level indicators of SES; hence, we used neighborhood-level poverty as a proxy. However, neighborhood SES indicators were shown to be a valid and reliable methodology [57]. Follow-up time was limited to an average of about 2 years due to limited study sampling time frame of 2007 through 2011. Utilizing administrative data to assess comorbidity may have caused us to misclassify some patients as not having comorbidity as they may have been diagnosed outside of the hospital/outpatient treatment purview. We also acknowledge the loss to follow-up in the dataset, as Floridians are known to be transient inhabitants; that is, they live in Florida for only a portion of the year, returning to other American climates or even other countries in the summer months, which may impact our ability to capture their utilization of health services and endpoints prospectively. Florida statistics on screenings are captured and produced by the Behavioral Risk Factor Surveillance System at the state and county levels and as of yet do not have figures at the census tract level. Also, our data were limited to the state of Florida which may not be generalizable to the rest of the United States population.

In summary, older CRC patients with more comorbidity, living in lower SES neighborhoods, without insurance, who are unmarried and diagnosed at later disease stage, are at higher risk of death. Higher risk among younger patients was associated with cancer site, receiving Medicaid services, and distant stage disease; however, lower SES and being unmarried were not risk factors in this age group. Targeted interventions to intervene to improve survivorship and greater social support while considering age group may assist these high-risk groups and warrant further study.
